# Targeted radioligand therapy in oncology: clinical development across targets and platforms

**DOI:** 10.3389/fonc.2026.1886049

**Published:** 2026-10-26

**Authors:** Jiaxin An, Xiaolei Meng, Lara Hathout, Ronald Ennis, Xinxin Zhang, Ning J. Yue, Matthew P. Deek, Salma K. Jabbour

**Affiliations:** 1 Rutgers Institute for Pharmaceutical Industry Fellowships, Ernest Mario School of Pharmacy, Rutgers University, Piscataway, NJ, United States; 2 Department of Radiation Oncology, Rutgers Cancer Institute, Rutgers Robert Wood Johnson Medical School, Rutgers University, New Brunswick, NJ, United States

**Keywords:** clinical trials, oncology, radioligand therapy, radiopharmaceuticals, targeted therapy

## Abstract

Radioligand therapy (RLT) has gained increasing prominence as an important therapeutic modality in oncology. By coupling a tumor-targeting ligand with a therapeutic radionuclide, therapeutic targeted RLT enables selective radiation delivery to a malignant site. Clinical validation of prostate-specific membrane antigen (PSMA)- and somatostatin receptor (SSTR)-directed therapies, together with the regulatory approvals of lutetium Lu 177 dotatate and lutetium Lu 177 vipivotide tetraxetan, has established proof of clinical utility for this approach and driven a broader expansion of RLT development across oncology. In this review, we outline the major emitter classes for therapeutic RLT, provide an overview of the active clinical development landscape with emphasis on different targeting strategies, discuss the toxicity considerations relevant to this modality, and conclude with a perspective on the major challenges and future directions shaping the field.

## 1 Introduction

Over the past decade, oncology drug development has increasingly shifted toward biomarker-informed and mechanism-based therapeutic strategies (1–3). In parallel with advances in targeted therapies, antibody-drug conjugates, immunotherapy, gene therapy, and cell-based approaches, radiopharmaceutical therapy has re-emerged as a clinically relevant treatment modality (4–9). Radioligand therapy (RLT) represents a form of precision oncology in which a ligand binds a tumor-associated target and delivers a therapeutic radionuclide directly to malignant tissue (10–12). Its therapeutic activity is mediated by targeted radionuclide delivery rather than intracellular release of a conventional cytotoxic payload (13–15). Therapeutic RLT can employ a range of tumor-targeting ligand formats, including small molecules, peptides, monoclonal antibodies, and engineered protein scaffolds (12). Small-molecule ligands generally exhibit rapid tumor penetration and systemic clearance and form the basis of established PSMA-directed therapies, whereas peptide-based approaches, including somatostatin analogs, combine receptor specificity with favorable pharmacokinetic properties and are well established in SSTR-directed therapy (16, 17). Antibody-based platforms provide high target specificity and prolonged tumor retention but are characterized by slower tissue penetration and blood clearance, which may require matching with longer-lived radionuclides (18). More recently, engineered protein scaffolds, including Affibody molecules, have emerged as compact alternatives to conventional antibodies, combining high-affinity target binding with rapid tissue penetration and clearance (19, 20). Together, these approaches illustrate the diversity of targeting platforms currently being developed for therapeutic RLT. Therapeutic RLT is also generally amenable to off-the-shelf administration, without the individualized manufacturing or ex vivo manipulation required for some advanced therapeutic platforms (21, 22). As an off-the-shelf therapeutic platform, RLT has emerged as a distinct and expanding area of oncology drug development within the broader landscape of targeted cancer therapeutics (23, 24).

Interest in therapeutic RLT has been driven not only by the clinical validation of target classes such as prostate-specific membrane antigen (PSMA) and somatostatin receptor (SSTR), but also by the broad expansion of development programs directed against Human Epidermal Growth Factor Receptor 2 (HER2), fibroblast activation protein (FAP), gastrin-releasing peptide receptor (GRPR), carbonic anhydrase IX (CAIX), delta-like ligand 3 (DLL3), folate receptor alpha (FRα), EGFR/cMET, and other tumor-associated antigens and receptors (23). As a result, the field now encompasses a broader range of radionuclides and ligand structures being developed across multiple disease settings, spanning established beta-emitting platforms as well as emerging alpha-emitter approaches and next-generation constructs (23, 25, 26).

RLT has progressed from a therapeutic concept to an increasingly active area of clinical development in oncology (27). Early studies established proof of concept that ligand-directed delivery of therapeutic radionuclides could achieve tumor localization and demonstrate antitumor activity, particularly in receptor-driven malignancies. More recent clinical progress has been most clearly demonstrated in biomarker-defined settings, particularly with PSMA- and SSTR-directed therapies, which have helped establish RLT as a clinically relevant therapeutic approach and expanded interest in the field (28, 29). To date, the U.S. Food and Drug Administration (FDA) has approved two therapeutic RLT products in oncology: lutetium Lu 177-dotatate (Lutathera^®^) for SSTR-positive gastroenteropancreatic neuroendocrine tumors (GEP-NETs) and lutetium Lu 177 vipivotide tetraxetan (Pluvicto^®^) for PSMA-positive metastatic castration-resistant prostate cancer (mCRPC) (12). Building on these milestones, a broader wave of clinical investigation is now evaluating therapeutic RLT across additional target classes and disease settings, while also refining ligand design, radionuclide selection, and treatment strategy (23). Despite this progress, key challenges remain in target and radionuclide selection, patient stratification, treatment sequencing, combination strategies, and toxicity management. The goal of this review is to provide a development-focused assessment of the therapeutic RLT landscape by integrating radionuclide characteristics with the clinical maturity of established and emerging molecular targets. In addition to reviewing alpha-, beta-, and auger-emitters, we systematically characterize the ongoing oncology clinical-trial landscape across target classes, radionuclides, clinical phases, and disease settings and examine how these patterns inform current development strategies. By linking the evolving clinical pipeline with toxicity, dosimetry, patient selection, treatment sequencing, and other translational considerations, this review aims to provide a broader framework for understanding where RLT development currently stands and the factors likely to shape its next phase in oncology.

## 2 Classification by radionuclide type

Radionuclide selection is a critical determinant in RLT, as key nuclear properties including physical half-life, decay mode, and type of emission directly govern emission energy, particle range in tissue, and linear energy transfer (LET), thereby shaping radiobiological effects and clinical behavior (17, 30–32). Differences in emission energy, range, and LET translate into distinct therapeutic characteristics, leading to variability in radiochemistry compatibility, *in vivo* stability, achievable specific activity, matching target biology, feasibility of dosimetry, and overall safety profile across RLT platforms (10, 33–35). An ideal therapeutic radionuclide for RLT combines (1) suitable emission characteristics; (2) a pharmacokinetically matched half-life; (3) stable radiochemistry with high specific activity; (4) practical dosimetry; and (5) an acceptable safety profile (36–38). On this basis, contemporary RLT drug development predominantly employs α-emitters, β-emitters, and Auger electron emitters, as well as radionuclides with mixed emission properties, with each class offering distinct advantages and limitations that inform indication selection, trial design, and therapeutic positioning (39–42).

### 2.1 Alpha emitters

A major limitation in clinical translation is the management of radioactive daughter nuclide recoil, which can result in unintended irradiation of radiosensitive organs such as the kidneys and salivary glands (40),. In addition, global availability of α-emitters remains constrained by complex production routes, limited reactor or accelerator capacity, and fragile supply chains, representing a critical bottleneck for large-scale clinical adoption (43, 44). Finally, uncertainties in α-particle dosimetry, combined with limited long-term safety data, continue to pose regulatory and clinical challenges, underscoring the need for standardized methodologies and prospective trials to define optimal use (45).

A key clinical advantage of α-emitters is their exceptionally high relative biological effectiveness, allowing effective tumor cell killing at low absorbed doses and potentially reducing the total administered activity required for therapeutic effect (46). The short path length of α-particles minimizes irradiation of surrounding normal tissues, which is particularly advantageous when targeting small lesions or disseminated single-cell disease (47). However, this same physical property renders α-therapy highly sensitive to targeting accuracy, as heterogeneous antigen expression or suboptimal tumor penetration can significantly limit efficacy (48).

Alpha-emitting radionuclides used in targeted radionuclide therapy are characterized by the emission of high-LET α-particles, typically depositing 50–230 keV/μm over a very short tissue range of approximately 50-100 μm, corresponding to only a few cell diameters (40, 41). This physical profile results in dense ionization tracks that induce complex, largely irreparable DNA double-strand breaks, leading to high cytotoxic efficiency that is relatively independent of cell cycle phase and oxygenation status (42). Clinically relevant α-emitters include ^223^Ra, ^225^Ac, ^212^Pb, ^212^Bi, ^211^At, and ^227^Th, each differing in half-life, decay chain complexity, and daughter radionuclide behavior, which strongly influences dosimetry and safety considerations (49, 50).

In 2013, the FDA approved the first α-emitting radiopharmaceutical, radium Ra 233 dichloride (Xofigo^®^), based on ALSYMPCA trial (NCT00699751), which demonstrated a survival benefit in patients with mCRPC with bone metastases, establishing proof-of-concept for targeted α-therapy in oncology (51). In the ALSYMPCA trial, 921 patients with mCRPC and symptomatic bone metastases were randomized to Xofigo (50 kBq/kg body weight every 4 weeks for 6 administrations by slow IV injections over 1 minute) or placebo, including both docetaxel-pretreated (57%) and docetaxel-naïve (43%) populations. Xofigo significantly prolonged overall survival (OS) compared with placebo in the overall intention-to-treat population (median 14.9 vs 11.3 months; hazard ratio [HR] 0.70, 95% CI 0.58–0.83; p<0.0001), with consistent benefit observed across docetaxel subgroups. Xofigo also delayed time to first symptomatic skeletal event in the overall population (median 15.6 vs 9.8 months; HR 0.66, 0.52–0.83; p=0.0004). Treatment was generally well tolerated, although higher rates of Grade 3–4 thrombocytopenia were observed in docetaxel-pretreated patients receiving Xofigo (52). The most frequently reported adverse events were bone pain (50% vs 62%), anemia (31% vs 31%), and nausea (36% vs 35%).

Besides ^223^Ra, ^225^Ac has also emerged as a highly promising alpha-emitter radioligand for therapeutic development (53). ^225^Ac-labeled PSMA ligands have demonstrated antitumor activity in heavily pretreated metastatic prostate cancer patients, including those resistant to β-emitting therapies (54). In the ongoing Phase I/II, open-label, multicenter study AcTION (NCT04597411), the safety, tolerability, and preliminary antitumor activity of ^225^Ac-PSMA-617 are being evaluated in PSMA-positive metastatic prostate cancer, using dose-escalation and expansion cohorts to inform dosing and assess early efficacy signals (53). In parallel, additional Phase II/III studies, including randomized evaluations of ^225^Ac-PSMA-617 versus standard therapies and dose-optimization trials of ^225^Ac-PSMA-I&T, are underway to more rigorously define clinical benefit (55).

Targeted α-therapy has also been explored in hematologic malignancies, such as leukemia and multiple myeloma (56, 57), as well as in solid tumors beyond prostate cancer, including neuroendocrine tumors (NET) and a range of other malignancies affecting the brain (58, 59), breast (60), lung (61), gastrointestinal tract (62), pancreas (63), ovary (64, 65), and urinary bladder (66), leveraging tumor-specific antigens and peptides for selective delivery (56, 67). Across tumor types, α-emitters are increasingly positioned either as salvage therapy following β-emitter failure or as potential early-line options for patients with low tumor burden, where their short range may offer maximal therapeutic benefit.

### 2.2 Beta emitters

β-emitting radionuclides have historically formed the backbone of targeted radionuclide therapy and represented the earliest clinically established class of radiotherapeutics (68). Compared with α-particles, β-particles exhibit lower LET and longer tissue penetration ranges, typically on the order of several millimeters, resulting in more diffuse energy deposition within tumors and surrounding tissues (69). This physical profile enables crossfire effects that can partially compensate for heterogeneous target expression and limited tumor penetration, but also increases irradiation of adjacent normal tissues, thereby constraining therapeutic indices in certain settings (70). Clinically relevant β-emitters include ^177^Lu, ^90^Y, ^131^I, ^153^Sm, ^89^Sr, and ^186/188^Re, each differing in half-life, β-particle energy, tissue penetration range, and associated γ emissions, which collectively influence dosimetry, therapeutic index, and organ-specific toxicity profiles (30, 71, 72).

The earliest FDA-approved therapeutic applications of β-emitting radiopharmaceuticals in oncology were largely focused on metastatic bone disease (73). Agents such as strontium-89 chloride (148 MBq by slow IV injection for 1–2 minutes, or a dose of 1.5-2.2 MBq/kg body weight) and samarium-153 lexidronam (^153^Sm-EDTMP, one time IV injection at 37 MBq/kg over 1 minute) were approved for the palliation of pain associated with osteoblastic bone metastases, particularly in advanced prostate and breast cancers (74, 75). While these agents are mechanistically distinct from ligand-directed RLT, these bone-seeking agents localize to areas of increased bone turnover and deliver β-particle radiation to metastatic lesions, achieving meaningful symptomatic relief. Nevertheless, their clinical benefit was limited to pain control, with minimal impact on tumor burden or overall survival, underscoring intrinsic limitations of β-emitter-based approaches for disease modification (75, 76).

Building on the earlier experiences with bone-targeting radionuclide therapies, the field has increasingly advanced toward ligand-directed RLT, in which a targeting vector binds tumor-associated receptors to achieve selective radiation delivery (12, 74, 77). In 2018, the FDA approved ^177^Lu-DOTATATE (Lutathera^®^) as the first ligand-directed RLT for the treatment of adult patients with somatostatin receptor–positive GEP-NETs, including foregut, midgut, and hindgut tumors. The approval was based on results from the NETTER-1 trial (NCT01578239) and supportive data from the ERASMUS Medical Center (MC) study (NCT00353093) (78, 79). NETTER-1 was a randomized, open-label phase III study that enrolled 229 patients with progressive, well-differentiated, locally advanced or metastatic somatostatin receptor-positive midgut neuroendocrine tumors, who were randomized 1:1 to receive Lutathera^®^ (7.4 GBq IV every 8 weeks for 4 cycles) plus long-acting octreotide 30 mg or high-dose long-acting octreotide 60 mg alone. For participants in Lutathera^®^ arm, concomitant IV amino acids were given with each administration for purpose of renal protection. Median progression-free survival (PFS) was not reached in the Lutathera arm compared with 8.4 months in the control arm, corresponding to a hazard ratio of 0.21. Most patients receiving Lu 177 dotatate completed treatment (77% received all four infusions), and although adverse events were common in both arms, treatment-related events occurred more frequently with Lu 177 dotatate (86% vs. 31%). The most common adverse events were nausea (59%) and vomiting (47%), largely linked to concurrent amino acid infusions and typically resolving afterward. Most other events were mild (Grade 1-2). Grade 3–4 adverse event rates were similar between groups, with some transient blood-related toxicities in the Lu 177 dotatate group but no observed kidney toxicity over the follow-up period (80). In the ERASMUS MC study, Lutathera was initially administered through an expanded-access peptide receptor radionuclide therapy (PRRT) protocol at a single site in the Netherlands and demonstrated an overall response rate (ORR) of 20%, providing additional clinical support for its efficacy in this population (79).

Following the success of Lutathera^®^, the FDA approved another β-emitting radioligands, ^177^Lu-PSMA-617 (Pluvicto^®^), for the treatment of adult patients with PSMA-positive mCRPC previously treated with androgen receptor (AR) pathway inhibition and taxane-based chemotherapy based on the phase III VISION trial (NCT03511664) in 2022 (81, 82). This open-label, randomized study enrolled 831 patients, who were randomized 2:1 to receive Pluvicto^®^ plus best standard of care (BSoC) or BSoC alone. Pluvicto^®^ demonstrated statistically significant improvements in the primary endpoints of overall survival (OS) and radiographic progression-free survival (rPFS). The hazard ratio (HR) for OS was 0.62 (95% CI: 0.52–0.74; p<0.001) for Pluvicto plus BSoC versus BSoC alone, with a median OS of 15.3 months (95% CI: 14.2–16.9) compared with 11.3 months (95% CI: 9.8–13.5), respectively. Patients receiving Pluvicto^®^ had a median exposure of 6.9 months with 5 treatment cycles, while standard care exposure lasted longer in the treatment group (7.6 vs. 2.1 months) with more cycles initiated compared to controls. The most frequently reported adverse events includes fatigue (43.1% vs 22.9%), dry mouth (38.8% vs 0.5%), nausea (35.3% vs 16.6%), and anemia (31.8% vs 13.2%) (81). Subsequent clinical development has progressively moved ^177^Lu-PSMA-617 into earlier treatment settings. The Phase III PSMAfore trial supported expansion of Pluvicto^®^ to patients with PSMA-positive mCRPC previously treated with an AR pathway inhibitor who were considered appropriate to delay taxane-based chemotherapy. More recently, the Phase III PSMAddition trial (NCT04720157) evaluated Pluvicto^®^ in combination with an AR pathway inhibitor and androgen deprivation therapy in patients with PSMA-positive metastatic hormone-sensitive prostate cancer (mHSPC), demonstrating a significant improvement in rPFS and supporting FDA approval in this setting in July 2026. Together, these developments mark the progressive expansion of PSMA-directed RLT from heavily pretreated mCRPC into earlier stages of metastatic prostate cancer.

Beyond these leading indications of somatostatin receptor–positive GEP-NETs and PSMA-positive mCRPC, β-emitter–based approaches are also being explored across a wider spectrum of solid tumors, including breast (83, 84), lung (85, 86), gastrointestinal (87–89), pancreatic (90), ovarian (91), and other malignancies (84), leveraging tumor-associated receptors, peptides, and small-molecule ligands for selective delivery. In modern oncology, β-emitting radiopharmaceuticals represent both a proven therapeutic mainstay and a critical benchmark for the continued evolution of the field (87, 92).

### 2.3 Auger emitters

Auger electron–emitting radionuclides represent a distinct class of therapeutic radiation sources characterized by extremely short-range, LET energy deposition at the nanometer scale (76). Unlike α- or β-particles, which deposit radiation over micrometer-to-millimeter distances, Auger electrons consist of cascades of low-energy electrons released following electron capture or internal conversion events (93). Their subcellular range, often comparable to the dimensions of DNA, results in highly localized ionization and dense radiation damage confined to the immediate vicinity of the decay site (94).

The therapeutic promise of Auger emitters is therefore uniquely dependent on intracellular and, in many cases, intranuclear localization (95). Because their cytotoxic effect is spatially restricted, Auger-emitting radiopharmaceuticals require highly efficient tumor targeting, cellular internalization, and retention near DNA to achieve meaningful tumor cell kill (10, 96). When positioned within or adjacent to the nucleus, Auger electrons can generate complex DNA double-strand breaks with radiobiological effectiveness approaching that of high-LET α-particles. However, when decay occurs at the cell surface or within extracellular compartments, the therapeutic impact is markedly diminished, highlighting a central delivery challenge that distinguishes Auger therapy from α- and β-based approaches (96).

Clinically relevant Auger emitters include ^125^I, ^111^In, ^67^Ga, ^123^I, and ^99m^Tc, each differing in half-life, electron emission spectra, and decay-associated photon emissions, which collectively influence dosimetry, imaging compatibility, and feasibility for therapeutic development (30, 76). Among these, several radionuclides such as ^111^In and ^125^I have long been used in diagnostic or imaging applications (97, 98). More recently, Auger emitters have attracted interest in oncology as next-generation payloads for highly specific tumor-targeting vectors (96), including small molecules (99), peptides (100, 101), antibodies (102–104), and conjugates incorporating nuclear localization sequences (NLS) (97, 105). Although no Auger-emitting radiopharmaceutical has yet received regulatory approval, several early-phase clinical studies have been conducted to explore their therapeutic potential across tumor types, including Phase 0 trial of ^161^Tb-DOTA-LM3 (106), Phase I study of ^111^In-DTPA-hEGF (107), and Phase I/II study of dual β+ Auger ^161^Tb-PSMA-I&T (108).

Taken together, Auger electron emitters represent an emerging and mechanistically unique modality within targeted radionuclide therapy. As radiopharmaceutical oncology continues to expand beyond established α- and β-based approaches, Auger emitters may provide a complementary avenue for next-generation radioligand therapies that operate at the level of subcellular targeting (109) and nanoscale therapeutic radiation delivery (110).

## 3 Clinical trials

The number of approved radioligand therapies in oncology remains limited (12). Currently, only approved RLT in this this space are Lutathera (^177^Lu-DOTATATE) and Pluvicto (lutetium Lu 177 vipivotide tetraxetan). Lutathera was the first RLT approved by European Medicines Agency (EMA) and FDA for SSTR-positive GEP-NET, which now has been approved in 40 countries worldwide (111). Pluvicto was approved for PSMA-positive mCRPC after androgen receptor pathway inhibitor (ARPI) therapy and taxane-based chemotherapy, and its indication was later expanded to include patients who have received ARPI therapy and are considered appropriate to delay taxane-based chemotherapy (82). For more details regarding the approved therapeutic RLT agents, see **Table 1**.

**Table 1:** Information on FDA approved radio ligand therapy products.

Drug	Target	Radio-nuclide	Approved indication	Approved year	Pivotal trial	Key efficacy results
Lutetium Lu 177 vipivotide tetraxetan (Pluvicto, Novartis Pharmaceuticals Corporation)	PSMA	^177^Lu	Adults with PSMA-positive mCRPC who have been treated with ARPI therapy and are considered appropriate to delay taxane-based chemotherapy.	2022	VISION (NCT03511664)	lutetium Lu 177 vipivotide tetraxetan with BSoC vs BSoC: Median rPFS, 8.7 months vs 3.4 (hazard ratio 0.40, [95% CI: 0.31, 0.52]; p-value <0.001). Median OS, 15.3 months vs 11.3 months (hazard ratio 0.62, [95% CI: 0.52, 0.74]; p-value <0.001).
			Adult patients with PSMA-positive mCRPC who have been treated with androgen receptor pathway inhibition and taxane-based chemotherapy.	2025	PSMAfore (NCT04689828)	lutetium Lu 177 vipivotide tetraxetan vs ARPI: Median rPFS, 9.3 months vs 5.6 (hazard ratio 0.41 [95% CI: 0.29, 0.56]; p-value <0.0001). Median OS, 24.5 months vs 23.1 months (hazard ratio 0.91 [95% CI: 0.72, 1.14]; p-value was not statistically significant).
Lutetium Lu 177 dotatate (Lutathera, Advanced Accelerator Applications USA, Inc., a Novartis company)	SSTR	^177^Lu	Adult and pediatric patients 12 years and older with SSTR-positive GEP-NETs, including foregut, midgut, and hindgut neuroendocrine tumors.	2024	NETTER-1 (NCT01578239)	Lutetium Lu 177 dotatate with octreotide LAR vs octreotide LAR Median PFS, NR vs 8.4 (hazard ratio 0.21 [95% CI: 0.13, 0.33]; p-value <0.0001).
					ERASMUS (NCT00353093)	ORR, 20% (95%CI: 18%, 23%) DoR, 19.4 months (range: 0, 95 months; 95% CI: 16.6, 23.7 months)

ARPI, androgen receptor pathway inhibitor; BSoC, best standard of care; GEP-NETs, gastroenteropancreatic neuroendocrine tumors; mCRPC, metastatic castration-resistant prostate cancer; NR, not reached; octreotide LAR, octreotide long-acting release; OS, overall survival; PFS, progression-free survival; PSMA, Prostate-specific membrane antigen; rPFS, radiographic progression-free survival; SSTR, somatostatin receptor.

Against this background, the clinical pipeline has expanded rapidly and now encompasses a broader range of investigational agents across different radionuclide classes, molecular targets, and indications. To define the clinical development landscape of RLT in oncology, we searched ClinicalTrials.gov using the intervention terms “radioligand” or “targeted radiotherapy” together with the condition terms “oncology”, or “cancer”, or “carcinoma”, respectively. Retrieved records were manually reviewed to confirm that each study had a primary treatment objective and involved targeted RLT directed against tumor-associated receptors, antigens, or other cell-surface targets. Diagnostic, imaging, and screening studies were excluded (**Figure 1**).

**Figure 1:**
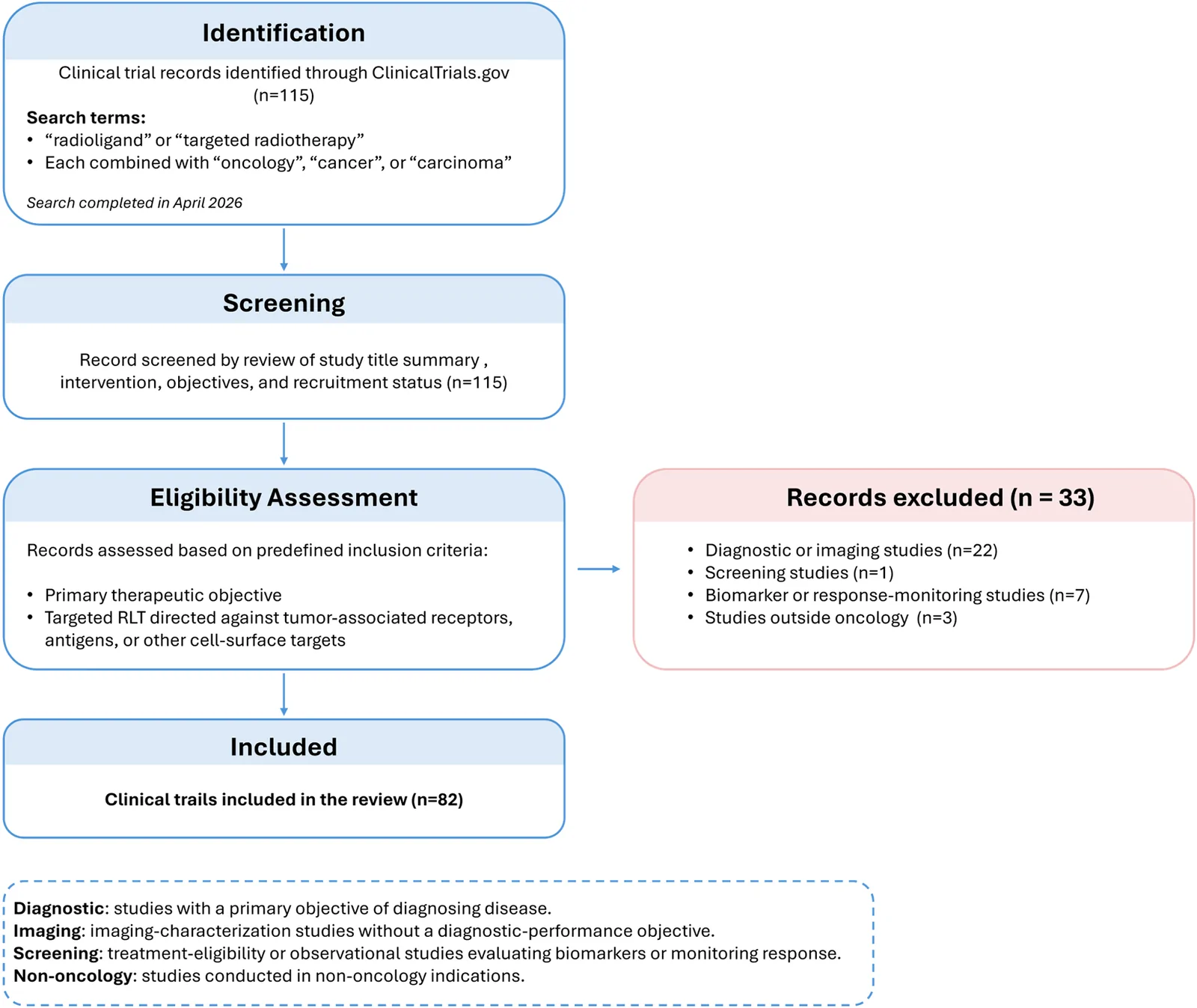
Flow diagram of clinical trial identification, screening, eligibility assessment, and inclusion. Selection of clinical trials identified through ClinicalTrials.gov as of April 2026 and principal reasons for exclusion.

As summarized in **Table 2**, key ongoing clinical trials highlight that current landscape is primarily concentrated in radioligands directed against targets such as PSMA, SSTR, HER2, and FAP. Radioligands directed against other tumor-associated antigens and receptors are also emerging in ongoing clinical development. The following sections discuss the ongoing clinical development of RLT across target classes. An overview of the key findings is provided in **Figure 2**.

**Table 2:** Representative of key ongoing trials based on target type.

Target	Emitter	Radionuclide	Phases	Conditions	NCT number
CAIX	beta	^177^Lu	Phase I	Carbonic Anhydrase IX -Expressing Solid Tumors	NCT05868174
			Phase III	Advanced Relapsed or Recurrent ccRCC	NCT07197580
CCK2R	beta	^177^Lu	Phase I	Thyroid Cancer, Medullary, Neuroendocrine Tumor of the Lung Grade 1 and 2, Neuroendocrine Tumor of the Thymus Grade 1 and 2, GEP-NET Grade 1-3	NCT02088645
CXCR4	alpha	^212^Pb	Early Phase I	Carcinoid Tumor Lung, Neuroendocrine Tumor of the Lung, Carcinoma, Small Cell Lung	NCT05557708
DLL3	alpha	^225^Ac	Phase I	Small Cell Lung Cancer, Large Cell Neuroendocrine Carcinoma of the Lung	NCT06736418
				Small Cell Lung Carcinoma, Large Cell Neuroendocrine Carcinoma of the Lung, Neuroendocrine Prostate Cancer, Gastroenteropancreatic Neuroendocrine Carcinoma	NCT07006727
		^212^Pb	Phase I/II	Large Cell Neuroendocrine Carcinoma, Large Cell Pulmonary Neuroendocrine Carcinoma of the Lung (LCNEC), Extrapulmonary Neuroendocrine Carcinoma (EP-NEC), Small Cell Lung Cancer (SCLC), Gastroenteropancreatic NEC, NEC of the Bladder, Other DLL3 Expressing epNEC	NCT07278479
EGFR/ cMET	alpha	^225^Ac	Phase I	Advanced Solid Tumor, Metastatic Colorectal Carcinoma, Head and Neck Squamous Cell Carcinoma, Non-small Cell Lung Cancer (NSCLC), Pancreatic Ductal Adenocarcinoma, Gastric Cancer, Renal Cell Carcinoma	NCT06147037
ER/PR+	beta	^177^Lu	Phase I	Locoregionally Recurrent Hormone-receptor Positive Breast Cancer, Metastatic Hormone-receptor Positive Breast Cancer	NCT07121244
FAP	beta	^177^Lu	Phase I/II	Solid Tumor	NCT04939610
		^177^Lu	Phase I	Pancreatic Ductal Adenocarcinoma, NSCLC, HR+/HER2- Ductal and Lobular Breast Cancer, Triple Negative Breast Cancer, Colorectal Cancer	NCT06562192
				FAP-Positive Tumors	NCT06636617
				Metastatic FAP-positive Solid Tumors	NCT06640413
				Ovarian Neoplasms, Breast Neoplasms, Pancreatic Intraductal Neoplasms, Colorectal Neoplasms, Esophageal Neoplasms, Stomach Neoplasms, Cholangiocarcinoma	NCT07213791
				Pancreatic Ductal Adenocarcinoma, NSCLC, HR+/HER2- Ductal and Lobular Breast Cancer, Triple Negative Breast Cancer, Colorectal Cancer, Soft Tissue Sarcoma	NCT07261631
		^90^Y	Phase II	NSCLC, Metastatic Lung Carcinoma	NCT07500285
GRPR	alpha	^212^Pb	Phase I	Cervical Cancer, Breast Cancer, Colon Cancer, NSCLC, mCRPC, Glioblastoma, NSCLC	NCT05283330
				Metastatic Colorectal Cancer	NCT07416552
	beta	^177^Lu	Phase I	Newly Diagnosed and Recurrent Glioblastoma	NCT05739942
				ER+, HER2- and GRPR+ Advanced Breast Cancer	NCT05870579
				Breast Neoplasms, Colorectal Neoplasms, Prostate Neoplasm, Endometrial Neoplasms, Neoplasm Metastasis	NCT07114601
			Phase I/II	Breast Cancer	NCT06247995
HER2	beta	^177^Lu	Early Phase I	Advanced Solid Tumors	NCT06824155
			Phase I	HER2-positive Metastatic Breast Cancer	NCT07081555
Integrin αvβ3	beta	^177^Lu	Phase I	Refractory Solid Tumor	NCT06375564
Integrin αvβ6	beta	^177^Lu	Phase I	Metastatic NSCLC	NCT06228482
PSMA	alpha	^225^Ac	Phase I	Prostatic Neoplasms, Castration-Resistant	NCT04597411
				Prostate Cancer, mCRPC, Oligometastatic Prostate Carcinoma, Hormone-Sensitive Prostate Cancer	NCT06229366
				mCRPC	NCT06217822
				mCRPC	NCT06879041
				Advanced Soft Tissue Sarcoma	NCT07156565
			Phase I/II	Prostate Cancer	NCT05983198
			Phase II	PSMA PET-Positive Castration-Resistant Prostate Cancer	NCT06549465
				mCRPC	NCT06909825
				mCRPC	NCT06402331
			Phase III	Prostate Cancer	NCT06855277
		^212^Pb	Phase I	CRPC, mCRPC	NCT07214961
	beta	^177^Lu	Phase I	Glioblastoma	NCT05109728
				Biochemically Recurrent Prostate Carcinoma, Prostate Adenocarcinoma	NCT06105918
				mCRPC	NCT06303713
				mCRPC	NCT06531499
				mCRPC	NCT07096128
			Phase I/II	CRPC, Colorectal Cancer, NSCLC, Ovarian Cancer, Cervical Cancer, Endometrial Cancer, TNBC, Triple Negative Breast Cancer, SCLC, Head & Neck Squamous Cell Carcinoma (HNSCC), Esophageal Squamous Cell Carcinoma (ESCC)	NCT07189871
				Extensive Stage SCLC	NCT05142696
				mCRPC	NCT05340374
			Phase II	Prostate Cancer	NCT04663997
				mCRPC	NCT07476001
				mCRPC	NCT07354594
				Prostate Cancer	NCT05114746
				Cancer of Unknown Primary	NCT07434180
				High Risk Localized Prostate Cancer Undergoing Radical Prostatectomy	NCT06798558
				Renal Cell Carcinoma, Advanced Clear Cell Renal Cell Carcinoma, Kidney Cancer	NCT06964958
				CRPC	NCT07047118
				Oligometastatic Prostate Cancer	NCT05560659
				Taxane Treatment Naive Chinese Male Patients with Progressive mCRPC	NCT05658003
				Recurrent Prostate Cancer	NCT06220188
				Prostate Cancer	NCT06259123
				Metastasis From Malignant Tumor of Prostate	NCT05204927
			Phase III	mCRPC	NCT06520345
				mCRPC	NCT04647526
				Prostatic Neoplasms	NCT04720157
				Metastatic Castration-Resistant Prostate Carcinoma, Metastatic Prostate Adenocarcinoma, Stage IVB Prostate Cancer AJCC v8	NCT07200830
				Prostate Cancer, Prostate Cancer (Diagnosis), High-risk Prostate Cancer, Localized Prostate Carcinoma, Very High risk Prostate Carcinoma	NCT07054346
	beta/alpha	^177^Lu/^225^Ac	Phase I	Prostate Cancer	NCT07381582
	beta	^161^Tb	Early Phase I	Castration-resistant Prostate Cancer	NCT06343038
			Phase I/II	Prostate Cancer, mCRPC	NCT05521412
			Phase I/II	Metastatic Neuroendocrine Prostate Cancer	NCT06379217
PSMA, SSTR2, GRPR	beta	^177^Lu	Phase I	Inoperable SSTR+ Well-differentiated GEP-NET That Has Progressed Following 177Lu-SSA Therapy	NCT05477576
SSTR	alpha	^255^Ac	Phase III	SSTR Positive Gastrointestinal Neuroendocrine Tumors, Pheochromocytoma/​Paragangliomas, Small Cell Lung, Renal Cell, and Head and Neck Cancers	NCT06479811
		^212^Pb	Phase I	Advanced SSTR2 Positive Neuroendocrine Tumors	NCT05636618
			Phase I/II	SSTR Positive, Gastrointestinal Neuroendocrine Tumors, Pheochromocytoma, Paragangliomas	NCT06427798
				Neuroendocrine Tumor	NCT05153772
			Phase II	SSTR2 Positive Neuroendocrine Tumors	NCT05249114
	beta	^177^Lu	Phase I	Metastatic Merkel Cell Carcinoma	NCT04261855
			Phase I/II	Metastatic Prostate Cancer That Has Neuroendocrine Cells	NCT05691465
			Phase II	Metastatic Unresectable Gastroenteropancreatic Neuroendocrine Tumors	NCT05773274
				Recurrent Meningioma	NCT06326190
				Intracranial Meningioma	NCT06955169
				SSTR Positive Advanced Bronchial Neuroendocrine Tumors	NCT04665739
				Neuroendocrine Tumors	NCT05387603
			Phase III	Neuroendocrine Tumors	NCT03049189
				GEP-NET	NCT03972488
				SSTR+, GEP-NET	NCT06784752
				Grade1–2 Advanced Midgut NETs	NCT06878664

CAIX, Carbonic Anhydrase IX; CCK2R, Cholecystokinin 2 Receptor; ccRCC, clear cell renal cell carcinoma; CRPC, Castration-Resistant Prostate Cancer; CXCR4, C-X-C chemokine receptor type 4; DLL3, Delta-like ligand 3; EGFR, Epidermal Growth Factor Receptor; ESCC, Esophageal Squamous Cell Carcinoma; EP-NEC, Extrapulmonary Neuroendocrine Carcinoma; ER, Estrogen Receptor; FAP, Fibroblast Activation Protein; GEP-NET, gastroenteropancreatic neuroendocrine tumor; GRPR, Gastrin-releasing peptide receptor; HER2, Human Epidermal Growth Factor Receptor 2; HNSCC, Head & Neck Squamous Cell Carcinoma; LCNEC, Large Cell Pulmonary; mCRPC, Metastatic Castration-resistant Prostate Cancer; NET, Neuroendocrine Tumor; NHSCC, Neck Squamous Cell Carcinoma; NSCLC, Non-small Cell Lung Cancer; PR; Progesterone Receptor; PSMA, Prostate-Specific Membrane Antigen; SCLC, Small Cell Lung Cancer; SSTR2, Somatostatin Receptor Type 2.

**Figure 2:**
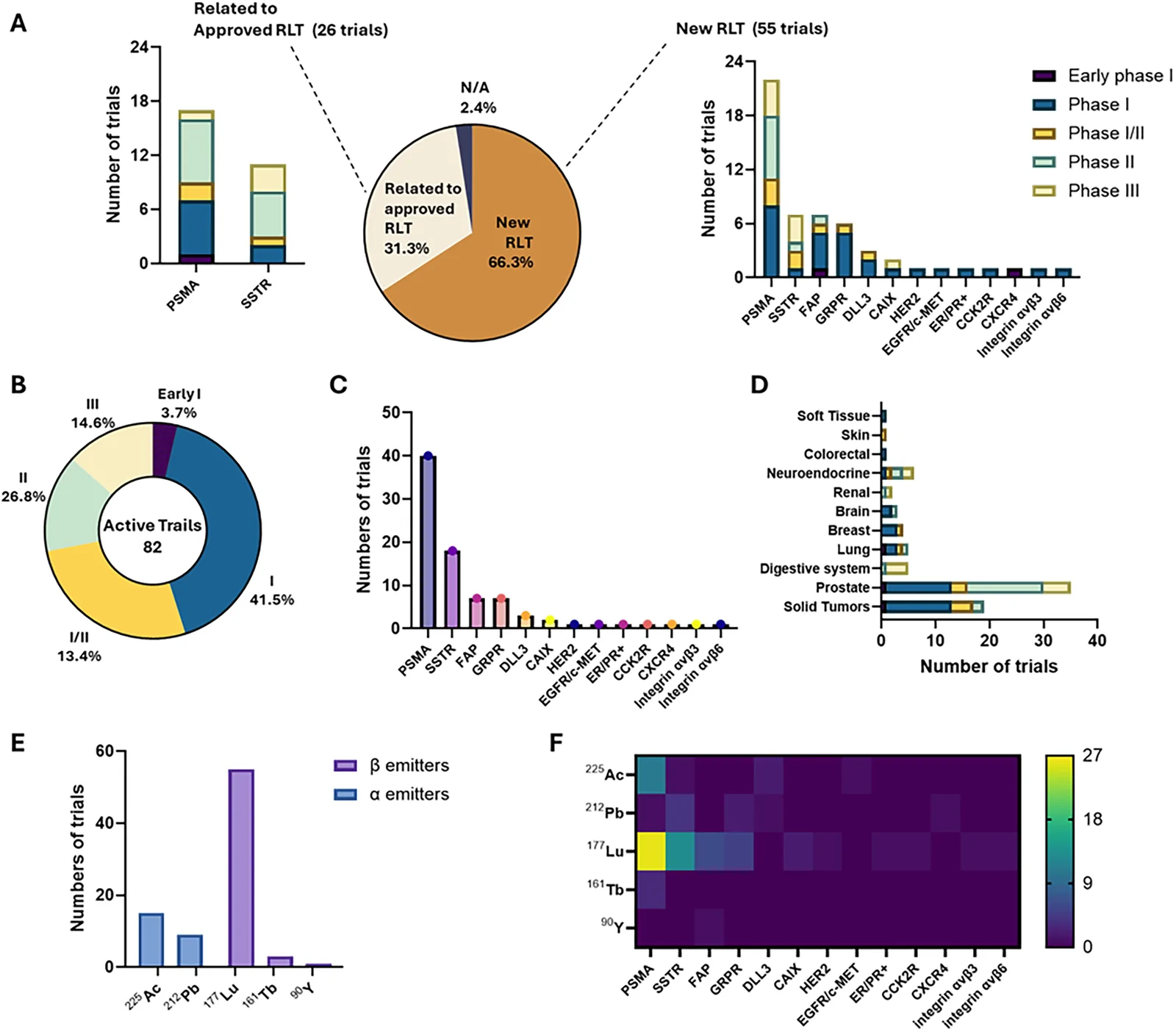
Overview of therapeutic targeted RLT in active oncology clinical trials. **(A)** Active trials associated with approved and investigational assets by target class and clinical phase. **(B)** Clinical phase composition of active trials. **(C)** Target classes under active clinical evaluation. **(D)** Tumor types under active clinical evaluation. **(E)** Radionuclides currently used in active trials by emitter class. **(F)** Heatmap of target class in relation to radionuclide types.

A total of 82 ongoing clinical trials has been identified in this review as of April 2026, including 3.7% in early Phase I, 41.5% in Phase I, 13.4% in Phase I/II, 26.8% in Phase II, and 14.6% in Phase III, indicating that the current RLT pipeline remains weighted toward early clinical development while already including a meaningful proportion of later-phase studies. These trials span a broad range of oncologic indications, including prostate cancer, neuroendocrine tumors, brain tumors, lung cancer, breast cancer, renal tumors, digestive system malignancies, colorectal cancer, skin malignancies, soft tissue tumors, and broader basket solid tumor populations (112, 113). Among defined disease-specific settings, prostate cancer is the leading indication and shows the most mature clinical development, with ^177^Lu-based radioligands accounting for most of the active trials, alongside additional expansion into ^225^Ac-, ^212^Pb-, ^161^Tb-, and combined ^177^Lu/^225^Ac-based approaches. Brain tumors represent a smaller but distinct area of development and, within the currently identified active trials, are represented by ^177^Lu-based programs. Lung cancer defines a more heterogeneous setting, with active development spanning ^177^Lu-, ^212^Pb-, ^225^Ac-, and ^90^Y-based radioligands (113). Abdominal malignancies are most clearly represented by digestive system and neuroendocrine indications, in which ^177^Lu-based radioligands account for most active trials, while additional development also includes ^225^Ac- and ^212^Pb-based approaches.

### 3.1 PSMA-targeted radioligands

PSMA-targeted RLT currently defines the most clinically advanced class of RLT in solid tumors (114). PSMA is a transmembrane glycoprotein that is highly expressed on the surface of prostate cancer cells, particularly in advanced and metastatic stages (115). The strong expression of PSMA in this setting, together with the ability of small-molecule ligands to achieve effective tumor targeting in metastatic disease, has made PSMA an attractive target for RLT development (116).

In the VISION trial (NCT03511664), ^177^Lu-PSMA-617 (7.4 GBq every 6 weeks for 4–6 cycles) improved both radiographic rPFS and OS in patients with PSMA-positive mCRPC (117). In PSMAfore trial (NCT04689828), ^177^Lu-PSMA-617 further demonstrated improved rPFS in taxane-naive patients with mCRPC (118). Together, both studies established PSMA as a clinically validated target in therapeutic RLT and supported continued expansion of this class into earlier disease settings.

Current PSMA-directed development is focused on extending beyond the initial late-line setting (119). Ongoing trials are evaluating PSMA-targeted radioligands earlier disease settings, including metastatic hormone-sensitive and localized high-risk prostate cancer. Additional studies are also evaluating PSMA-targeted radioligands in combination with chemotherapy and other systemic therapies.

In parallel with the progress achieved by beta-emitting PSMA-targeted radioligands, several alpha-emitting agents are also advancing through ongoing clinical development in prostate cancer. AcTION (NCT04597411) is a Phase I study evaluating ^225^Ac-PSMA-617 in patients with PSMA-positive prostate cancer with or without prior exposure to ^177^Lu-PSMA radioligand therapy, reflecting continued interest in actinium-based therapy both before and after beta-emitting RLT (120). AlphaBreak (NCT06402331) is an open-label randomized study of FPI-2265 (^225^Ac-PSMA-I&T) in PSMA-positive mCRPC (121), and NCT06909825 is further extending this platform by evaluating FPI-2265 in combination with olaparib in mCRPC. In parallel, SatisfACtion (NCT05983198) is a Phase I/II study of ^225^Ac-PSMA-R2 in metastatic hormone-sensitive prostate cancer (mHSPC) and mCRPC, expanding alpha-emitting PSMA-directed development beyond late-stage clinical setting. PAnTHA (NCT06217822) is a first-in-human Phase I study of ^225^Ac-PSMA-Trillium in advanced mCRPC, adding another actinium-based RLT to the current clinical landscape (122). Beyond ^177^Lu- and α-emitting approaches, terbium-161 (^161^Tb) is emerging as an alternative radionuclide for PSMA-directed RLT. ^161^Tb emits β-particles with characteristics similar to ^177^Lu but additionally produces short-range Auger and conversion electrons, which may increase radiation deposition in small tumor deposits and micrometastases. The VIOLET trial (NCT05521412) is a single-center, Phase I/II study evaluating [^161^Tb] Tb-PSMA-I&T in patients with mCRPC. First-in-human results demonstrated that treatment at an administered activity of 7.4 GBq was feasible and associated with relatively few Grade 3–4 treatment-related adverse events, together with encouraging preliminary antitumor activity (123–125). These findings provide early clinical evidence supporting further development of ^161^Tb as a next-generation radionuclide for PSMA-targeted RLT.

Taken together, the current alpha and beta-emitter trial landscape shows that PSMA-directed RLT development is no longer focused solely on demonstrating activity in heavily pretreated disease. Instead, ongoing studies are addressing several parallel clinical questions, including whether alpha-emitting radioligands can provide activity after prior ^177^Lu-PSMA exposure, whether they can be introduced earlier in the disease course, and whether combination strategies can further improve therapeutic outcomes. The range of RLT now under evaluation reflects active optimization at the level of radionuclide choice and ligand scaffold (118). Overall, current development of PSMA-directed RLT is focused not only on demonstrating clinical benefit, but also on treatment sequencing, combination strategies, and radionuclide optimization.

### 3.2 SSTR-targeted radioligands

SSTR-targeted radioligands represent one of the earliest and most established applications of targeted radionuclide therapy in oncology, particularly in treating NETs (28). High and sustained SSTR expression across well-differentiated NETs has enabled effective tumor targeting and imaging with radiolabeled somatostatin analogs, supporting the development of PRRT as a standard treatment option in advanced disease (28).

Clinically, in NETTER-1 trial (NCT01578239), ^177^Lu-DOTATATE, have demonstrated significant improvements in progression-free survival and durable tumor control in patients with advanced gastroenteropancreatic NETs across multiple prospective studies (80, 126). These findings have supported global regulatory approvals and widespread adoption in later-line treatment settings.

More recently, SSTR-targeted RLT have advanced into earlier-line treatment settings and broader clinical development. The Phase III study NETTER-2 (NCT03972488) was the first study to report results for RLT in first-line setting in patients across all cancer types (127). In this randomized trial, ^177^Lu-DOTATATE in combination with octreotide LAR was shown to significantly prolonged PFS in Grade 2 and 3 advanced gastroenteropancreatic NETs, establishing the benefit of SSTR-targeted RLT in earlier treatment settings.

In parallel, there are ongoing studies that are also investigating retreatment strategies, lung NET-specific applications (128), neoadjuvant use (129), and alternative radionuclides such as ^225^Ac- and ^212^Pb-labeled somatostatin analogs (130–133) to improve therapeutic index and overcome resistance. The development of SSTR-targeted RLT is passing through the validation phase and entering into the phase of exploring line-of-therapy expansion, optimizing patient selection, radionuclide innovation, and refinement of therapeutic index.

### 3.3 GRPR-targeted radioligands

GRPR has emerged as another therapeutic target of interest because of its expression across multiple solid tumors, including prostate cancer, breast cancer, and glioblastoma (134). In contrast to PSMA and SSTR, GRPR-targeted RLT remains at an earlier stage of clinical development. A growing range of GRPR-directed radiopharmaceutical platforms has been developed, including both agonist- and antagonist-based ligands and constructs labeled with therapeutic radionuclides such as lutetium-177.

Among currently developing GRPR-targeted therapeutic radioligands, ^177^Lu-NeoB represents the most prominent ongoing clinical program. NeoRay (NCT03872778) is a first-in-human Phase I/IIa trial evaluating ^177^Lu-NeoB in patients with advanced solid tumors with GRPR overexpression (135). Additional disease-specific studies are extending ^177^Lu-NeoB treatment into combination-based settings. In a Phase I dose finding trial (NCT05739942), ^177^Lu-NeoB is being evaluated in combination with radiotherapy and temozolomide in newly diagnosed glioblastoma (136). In another Phase I trial (NCT05870579), ^177^Lu-NeoB is being evaluated in combination with ribociclib and fulvestrant in patients with ER-positive, HER2-negative, GRPR-positive advanced breast cancer. Collectively, the current ^177^Lu-NeoB clinical program indicates that GRPR-directed RLT remains focused mainly on early-Phase therapeutic development, with emphasis on identifying suitable disease settings, defining feasible combination partners, and establishing preliminary evidence of antitumor activity.

### 3.4 HER2-targeted radioligands

As a well-established therapeutic target in oncology, HER2-directed RLT is conceptually attractive, especially in breast and gastroesophageal cancers (12). Representative studies include NCT04467515, a dose-escalation and dose-expansion study of CAM-H2 in advanced or metastatic HER2-positive breast, gastric, and gastroesophageal junction cancers; NCT06824155 (HEAT), a first-in-human Phase 0/1 study of ^177^Lu-RAD202; and NCT07081555, a first-in-human Phase I study of [^177^Lu]Lu-ABY-271, an Affibody-based HER2-targeting radioligand, in HER2-positive metastatic breast cancer. These programs illustrate the range of molecular targeting approaches currently being evaluated for HER2-directed radionuclide therapy. Collectively, HER2 programs remain early with multiple Phase I trial ongoing which are still focused largely on feasibility, dosimetry, and activity-signal generation rather than definitive efficacy testing.

The development of HER2-targeted RLT is also facing several challenges. HER2-targeted therapies have already been embedded within a well-established treatment landscape, with approved agents including monoclonal antibodies, tyrosine kinase inhibitors and antibody-drug conjugates (ADCs) (137, 138). Accordingly, given the potential addition of radioligand-related toxicity, HER2-targeted RLT needs to demonstrate meaningful therapeutic improvement beyond existing HER2-directed options to support a positive benefit-risk ratio and to become established as a treatment option (139). This challenge is particularly significant in patients with pretreated HER2-expressing disease, where primary and acquired resistance to HER2-directed therapies is well recognized. In conclusion, compared with PSMA and SSTR, HER2-directed RLT is viewed as a target class with strong biological rational but limited clinical maturity, and its future role will depend on whether early-Phase studies can define a clear therapeutic niche within the broader HER2-directed treatment landscape.

### 3.5 FAP-targeted radioligands

FAP has recently gained interest as a pan-tumor target due to the widespread expression of FAP in cancer-associated fibroblasts across a broad range of epithelial and mesenchymal malignancies (139). Unlike many tumor cell-restricted targets, FAP is predominantly expressed within the tumor stroma, enabling high tumor-to-background contrast and broad applicability across tumor types (140, 141). Early clinical studies of FAP-targeted PET agents have demonstrated rapid tumor uptake, low background signal, and consistent visualization of diverse malignancies, supporting their use in tumor detection, staging, and radiotherapy planning (141).

Therapeutically, both beta- and alpha-emitting FAP-targeted radioligands are being evaluated in early-Phase clinical trials across multiple solid tumors, including sarcoma, pancreatic, breast, and colorectal cancers. In a case series, early clinical experience with ^90^Y-FAPI-46 showed that FAP-targeted therapy was feasible and generally well tolerated, though clinical efficacy data remains limited and heterogeneous across tumor types (142).

More recent development has shifted toward agents designed to improve tumor retention and therapeutic potency, including ^177^Lu-FAP-2286, a peptide-based FAP-targeting radioligand; ^177^Lu-LNC1004, an Evans blue-modified FAP inhibitor designed to prolong tumor retention; and ^177^Lu-OncoFAP-23, a multivalent derivative of the high-affinity small-molecule OncoFAP platform. Overall, current FAP-directed RLT development remains early and is focused mainly on optimizing ligand design, improving dosimetry, and identifying patient populations.

### 3.6 Other emerging target classes

Several additional target classes remain at an early stage of therapeutic RLT development. DLL3-directed RLT is being explored predominantly in neuroendocrine malignancies and other DLL3-positive solid tumors, with early clinical programs evaluating different targeting formats, including macrocyclic peptide- and VHH-Fc–based agents labeled with the alpha-emitter ^225^Ac (143, 144). FRα-directed RLT development includes ^177^Lu-EVS459, a low-molecular-weight FR-targeting radioligand paired with a ^68Ga-labeled imaging counterpart and is being investigated mainly in ovarian cancer and selected lung cancers, largely in Phase I therapeutic programs (12, 145, 146). GFR/cMET-directed RLT is represented by ^225^Ac-FPI-2068, an alpha-emitting radioimmunoconjugate based on a bispecific antibody targeting both EGFR and cMET, currently in early clinical development across several solid tumors (147, 148). In parallel, CEA-directed RLT development has employed a pretargeted radioimmunotherapy strategy in which tumor localization by a CEA-targeting bispecific antibody is followed by administration of a ^177^Lu-labeled peptide, with clinical experience reported particularly in colorectal cancer (149–151). These targets illustrate that a portion of the current RLT development landscape is still dominated by early trials including first-in-human and proof-of-concept studies.

Current therapeutic RLT development is distributed across target classes with a broad range of clinical maturity (12). PSMA- and SSTR-directed RLT represent the most clinically advanced programs and shed light on target validation, regulatory translation, and treatment integration (152–154). HER2-, FAP-, GRPR-, CAIX-, DLL3-, FRα-, EGFR/cMET-, and CEA-directed programs remain earlier in clinical development and are still being evaluated across different indications, ligand formats, and treatment strategies (1, 12). Together, these programs illustrate the growing breadth of target exploration in therapeutic RLT and support continued development across diverse tumor types, disease stages, and treatment settings in oncology.

## 4 Toxicities of radioligand therapy

RLT has emerged as a highly effective targeted treatment modality for several malignancies, most notably neuroendocrine tumors (NETs) and mCRPC, by delivering cytotoxic radiation directly to tumor cells via tumor-specific ligands. Despite its favorable therapeutic index compared with conventional systemic therapies, RLT is associated with a distinct spectrum of treatment-related toxicities arising from both on-target and off-target radiation exposure, most commonly affecting the bone marrow, kidneys, and salivary glands (119, 155, 156). As the clinical use of β- and α-emitting radioligands continues to expand, including earlier lines of therapy and combination regimens, understanding the incidence and nature of these adverse effects has become increasingly important. Moreover, effective toxicity management strategies, such as dose optimization, organ-protective measures, standardized toxicity grading, and long-term surveillance, are critical to maximizing therapeutic benefit while minimizing harm and improving patients’ quality of lives. This section will focus on the current evidence on RLT-associated toxicities and their management, with a focus on clinical patterns, risk factors, and emerging approaches to improve treatment safety. Refer to **Table 3** for a summary of comparative toxicities and risk-mitigation strategies for therapeutic RLT.

**Table 3:** Comparative toxicities and risk-mitigation strategies for therapeutic radioligand therapy.

Organ system/toxicity	β-emitting therapies	α-emitting therapies	Major risk factors	Risk-mitigation strategies
Anemia, thrombocytopenia, leukopenia or neutropenia	With ^177^Lu-DOTATATE and similar agents, any-Grade hematologic toxicity occurs in approximately 10–25%, and Grade 3–4 events generally occur in ≤10–15%. With ^177^Lu-PSMA, Grade 3–4 anemia occurs in approximately 7-12%, with lower rates of severe thrombocytopenia and neutropenia.	Comparative rates remain less well defined because evidence is based mainly on small and heterogeneous studies. Marrow toxicity may occur despite the short range of α-particles, particularly in patients with extensive skeletal disease or impaired marrow reserve.	Baseline cytopenias; extensive bone metastases; previous chemotherapy or radiotherapy; high cumulative activity; reduced marrow reserve.	Baseline and serial complete blood counts; appropriate treatment intervals; individualized dose adjustment; treatment delay or discontinuation for persistent cytopenias; transfusion or growth-factor support when indicated
Myelodysplastic syndrome or leukemia	Reported in approximately 0.5-2% of peptide receptor radionuclide therapy cohorts with extended follow-up.	Long-term incidence remains uncertain because of limited follow-up and smaller patient populations.	Previous alkylating chemotherapy; prior radiotherapy; cumulative marrow exposure; persistent cytopenias.	Long-term hematologic surveillance; investigation of prolonged unexplained cytopenias; minimization of cumulative marrow exposure.
Decline in renal function or nephrotoxicity	Severe renal toxicity was reported in up to 14% with earlier high-dose ^90^Y-based therapy without adequate renal protection. With contemporary ^177^Lu-DOTATATE, Grade 3–4 toxicity is generally <1-2%. Clinically meaningful injury is also uncommon with ^177^Lu-PSMA.	Rates are not well established. High linear energy transfer may cause substantial injury when α-emitting agents or daughter radionuclides are retained in renal tissue.	Pre-existing kidney disease; hypertension; diabetes; older age; urinary obstruction; dehydration; nephrotoxic treatment; high absorbed renal dose.	Baseline and serial renal-function testing; amino-acid infusion with lysine and arginine for peptide-based therapy; hydration; correction of urinary obstruction; avoidance of nephrotoxic agents; individualized renal dosimetry.
Salivary glands: xerostomia	Grade 1–2 xerostomia occurs in approximately 20-40% with ^177^Lu-PSMA; severe events are uncommon.	Xerostomia occurs in approximately 60–70% or more with ^225^Ac-PSMA and may be dose limiting.	High physiologic salivary-gland uptake; high cumulative activity; repeated treatment cycles; α-emitter use.	Dose individualization or de-escalation; longer treatment intervals; tandem α/β strategies; hydration; gland cooling; sialagogues; investigation of radioprotective agents and modified ligands.

### 4.1 Hematologic toxicity

Hematologic toxicity represents one of the most frequently observed adverse effects of RLT (157). The toxicity primarily attributes to radiation exposure of the bone marrow from circulating radioligands and crossfire effects (158). Across β-emitting therapies such as ^177^Lu-labeled compounds, transient cytopenias, which including anemia, thrombocytopenia, and leukopenia, are commonly reported, with any-Grade hematologic toxicities occurring in roughly 10-25% of patients receiving ^177^Lu-DOTATATE PRRT and similar agents, and Grade 3–4 events typically occurring in ≤10-15% of individuals, most often in settings of prior marrow compromise or extensive bone metastases (157, 159, 160). In mCRPC treated with ^177^Lu-PSMA therapies, pooled analyses suggest Grade 3–4 hematologic adverse events such as anemia occur in about 7-12% of patients with thrombocytopenia and neutropenia generally reported at lower percentages (157, 161). Higher-Grade events are more likely in patients with baseline cytopenias, previous chemotherapy, or high bone tumor burden (157).

In addition to acute and subacute cytopenias, rare but clinically significant long-term sequelae such as therapy-related myelodysplastic syndromes or leukemia have been reported at low rates (0.5-2%) in PRRT cohorts with extended follow-up (159). Importantly, hematologic toxicities are generally manageable with routine laboratory monitoring, supportive care, and interval adjustments, and most patients recover to baseline, or near-baseline counts following treatment completion (162).

### 4.2 Renal toxicity

Renal toxicity is a recognized concern in RLT because kidneys participate in glomerular filtration and proximal tubular reabsorption of radiolabeled peptides, which can lead to prolonged radiation exposure of renal tissue and potential functional decline (163). Severe nephrotoxicity (CTCAE Grade 3–4 or higher) has historically been reported in up to 14% of patients, particularly with earlier high-dose ^90^Y-based PRRT without renal protection, though modern ^177^Lu-based therapies show markedly lower rates of clinically significant renal injury (164). Renal-protective strategies such as co-infusion of positively charged amino acids (e.g., lysine/arginine) substantially reduce renal uptake of radiopeptides and mitigate radiation dose to the kidneys (165). In contemporary ^177^Lu-DOTATATE PRRT cohorts, severe renal toxicity is rare (often <1-2%), with most patients experiencing only mild, transient changes in creatinine or estimated GFR and no Grade 3–4 nephrotoxicity in many series (166). Similarly, ^177^Lu-PSMA therapies for prostate cancer infrequently cause clinically meaningful renal impairment, even in patients with pre-existing renal dysfunction, and prospective data generally show stable or minimally changed GFR after treatment (167). Risk factors for renal toxicity include older age, baseline chronic kidney disease, hypertension, diabetes, prior nephrotoxic chemotherapy, and higher absorbed renal doses (167, 168). Tailored dosimetry and renal-protective measures remain critical to minimizing renal risks while preserving therapeutic benefit (169).

### 4.3 Xerostomia (PSMA-specific)

Xerostomia, or dry mouth due to salivary gland dysfunction, is a clinically relevant toxicity of PSMA-targeted radioligand therapies because PSMA ligands bind nonspecifically to salivary gland tissue and deliver radiation there (119).

In patients treated with ^177^Lu-PSMA-I&T/-617, mild-to-moderate xerostomia (CTCAE Grade 1-2) has been observed in approximately 20 to 40% of patients during short- and longer-term follow-up (170). In many cohorts using ^177^Lu-PSMA alone, only minor salivary gland toxicity has been observed whereas higher-Grade xerostomia is uncommon (171). Retrospective clinical data also showed variable Grade 1–2 xerostomia, with earlier phase studies reporting up to 24% and others noting a phase III prevalence of 38-39% in ^177^Lu-PSMA-617 therapy cohorts (170). By contrast, α-emitter therapies such as ^225^Ac-PSMA are associated with a higher incidence and severity of xerostomia, often becoming a dose-limiting toxicity; pooled analyses estimate xerostomia in 60 to 70% or more of patients receiving ^225^Ac-PSMA treatments (172). The pathophysiology is largely attributed to off-target uptake of PSMA ligands in salivary glands, leading to radiation-induced acinar damage (173). Various protective approaches have been explored, such as gland cooling, oral hydration, or sialagogues, which remain as consistent and effective mitigation strategies under investigation (174).

## 5 Discussion and conclusion

Therapeutic RLT has become an increasingly important treatment platform in oncology, with clinical development now extending across multiple target classes, ligand structures and radionuclide types (24). PSMA- and SSTR-directed radioligands currently represent the most clinically advanced programs, while ongoing development directed against HER2, FAP, GRPR, CAIX, DLL3, FRα, EGFR/cMET, CEA, and other targets continues to broaden the field. At the same time, therapeutic RLT is no longer defined only by established beta-emitting platforms but increasingly includes alpha-emitting and other emerging radionuclide strategies that are expanding the clinical ambition of the field (23). Despite this progress, important limitations and additional considerations remain to be addressed as therapeutic RLT continues to evolve in oncology.

### 5.1 Dosimetry, cumulative exposure, and treatment optimization

One of the unresolved issues in therapeutic RLT remains dosimetry. The biological effect of RLT is shaped not only by target expression, but also by radionuclide decay properties, tumor residence time, organ distribution, intratumoral heterogeneity, and cumulative exposure to normal tissues. These factors become particularly important as development extends into earlier lines of therapy, retreatment strategies, sequential radionuclide approaches, and combination regimens, where patients may receive repeated courses of radioligand exposure over a longer course of treatment. In this context, the balance between efficacy and tolerability cannot be understood solely at the level of a single cycle or a single regimen.

One of the current attempts in tacking the dosimetry issues is to utilize single-photon emission computed tomography (SPECT) to establish 3D distributions of radionuclide inside patient anatomic organs so that more accurate 3D dosimetric distributions can be computed and established (175). Most of the above-mentioned radionuclides emit not only the particles used for therapeutic purposes but also photons which have high enough energies to allow for SPECT imaging (176). Active research has been conducted in this area, and several commercial systems are now available for clinical use (177).

Building on imaging-based dose assessment, personalized dosimetry may provide an approach to tailoring treatment according to differences in radioligand uptake and clearance across individual patients. Such approaches generally use quantitative imaging to estimate radiation exposure in tumors and normal organs and may support adjustment of administered activities, treatment intervals, and the treatment cycles (178). More detailed methods, including voxel-based analysis, may also help characterize spatial heterogeneity in radiation delivery (179). In principle, personalized dosimetry approaches could support treatment optimization. Treatment intensification may be considered when tumor uptake is favorable and the predicted absorbed doses to normal organs at risk remain acceptable (180). Conversely, elevated predicted doses to normal organs at risk may support dose reduction or other treatment modifications, thereby improving the balance between therapeutic benefit and toxicity. Prospective studies have demonstrated the feasibility of this individualized approach. In the P-PRRT trial, the administered activity of subsequent cycles of ^177^Lu-octreotate was adjusted according to renal function and the kidney absorbed dose per unit of administered activity observed during the preceding cycle. The ongoing randomized Phase III DUONEN trial directly compared a conventional fixed-activity regimen with several dosimetry-guided PRRT strategies. However, the initial P-PRRT results primarily established the feasibility and safety of personalized administration, whereas the currently reported DUONEN findings are limited to an interim safety analysis. Thus, although these studies provide important prospective support for individualized treatment, definitive evidence that dosimetry-guided administration improves clinical efficacy relative to fixed-activity regimens remains unavailable. Personalized dosimetry may therefore represent an important direction for future treatment optimization, but its clinical role will need to be established prospectively for specific radioligands, treatment regimens, and disease settings.

Regulatory expectations are increasingly emphasizing prospective dosage optimization rather than reliance on a single administered activity selected primarily from early safety experience (181). In 2025, the FDA issued the draft guidance for industry, Oncology Therapeutic Radiopharmaceuticals: Dosage Optimization During Clinical Development, to provide recommendations on dosage optimization during clinical development. Although this guidance is not intended to address the choice between fixed administered activity dosing and dosing determined by personalized dosimetry, it outlines key considerations for establishing an appropriate dosage regimen for therapeutic radiopharmaceuticals. Specifically, the FDA recommends that sponsors identify an optimized dosage, defined as the administered activity and schedule, supported by adequate characterization of dose-response relationships and evaluation of the safety and activity associated with repeated treatment cycles. European and international initiatives similarly support more systematic characterization of absorbed dose, while allowing the specific approach to depend on the radiopharmaceutical and its intended clinical use. In Europe, the EMA’s 2024 Concept Paper on the Clinical Evaluation of Therapeutic Radiopharmaceuticals in Oncology identifies dosimetry and absorbed-dose assessment among the key points to be addressed in the future guideline on the clinical evaluation of therapeutic radiopharmaceuticals in oncology. Taken together, the recent FDA and EMA initiatives indicate growing regulatory attention to dosage and dosimetry optimization in the clinical development of RLT, while the specific evidentiary and methodological expectations for implementing personalized dosimetry remain to be established.

The transition from investigational dosimetry to routine individualized treatment is also limited by substantial methodological and operational variability (182). Absorbed-dose estimates can be influenced by the timing and number of imaging assessments, reconstruction and registration methods, delineation of tumors and normal organs, kinetic modeling, and user-dependent analysis (183). Variation in these components across clinical sites may reduce the comparability of absorbed-dose estimates and complicate the implementation of dosimetry in multicenter trials. Broader implementation will therefore require harmonized acquisition and analysis procedures, appropriate quality-control programs, and transparent characterization of measurement uncertainty. Existing clinical experience, together with recent health authority guidance and concept paper, provides an initial framework for developing and implementing personalized dosimetry in RLT. However, additional multicenter validation, software benchmarking, automation, and personnel training will be needed to support reproducible use at scale.

As therapeutic RLT continues to expand, cumulative radiation burden to marrow, kidneys, salivary glands, and other organs may become an important constraint on long-term treatment feasibility. This is especially relevant for high-potency radionuclide approaches and for treatment sequences that include prior external-beam radiotherapy, cytotoxic chemotherapy, or other marrow-stressing therapies. A major future need will therefore be the transition from largely fixed-activity paradigms toward more biologically informed, exposure-aware, and patient-specific treatment approaches. Cumulative exposure assessment may also become increasingly important for trial eligibility, treatment sequencing, and long-term clinical implementation.

### 5.2 Overall conclusions

Overall, the current clinical development landscape remains highly active but still requires substantial validation. The growing range of target classes, ligand formats, and radionuclide platforms further supports continued expansion of the field and highlights the potential for future therapeutic development beyond currently established indications. Favorable nonclinical or early clinical findings do not always translate into meaningful clinical benefit, and whether early signals of success can be reproduced in later-phase development remains uncertain, particularly given the heterogeneity of RLT design across radionuclides, ligand structures, and dosing strategies. How future clinical trials address these limitations and define the role of therapeutic RLT across different tumor settings will be critical to the continued evolution of the field and remains an area of considerable interest.

Taken together, therapeutic RLT has emerged as an increasingly important treatment modality in oncology, supported by clinically validated target classes and a rapidly expanding development landscape. Although important challenges remain, including unresolved dosimetry questions, complexity in clinical trial design and validation, and ongoing concerns related to toxicology and long-term safety, therapeutic RLT continues to demonstrate broad translational potential across multiple tumor types and treatment settings. With its distinct mechanism of target-directed radiation delivery, therapeutic RLT supports applications in imaging, staging, and patient selection, is now increasingly integrated into oncology treatment including movement into earlier lines of therapy and may ultimately serve as a broader platform for theranostic development.
